# Berlin Squirrelpox Virus, a New Poxvirus in Red Squirrels, Berlin, Germany

**DOI:** 10.3201/eid2310.171008

**Published:** 2017-10

**Authors:** Gudrun Wibbelt, Simon H. Tausch, Piotr W. Dabrowski, Olivia Kershaw, Andreas Nitsche, Livia Schrick

**Affiliations:** Leibniz Institute for Zoo and Wildlife Research, Berlin, Germany (G. Wibbelt);; Robert Koch Institute, Berlin (S.H. Tausch, P.W. Dabrowski, A. Nitsche, L. Schrick);; Free University Berlin, Berlin (O. Kershaw)

**Keywords:** squirrelpox, poxvirus, squirrel, Sciuridae, Berlin, Germany, skin disease, dermatitis, viruses, red squirrels, Sciurus vulgaris, Berlin squirrelpox virus

## Abstract

Near Berlin, Germany, several juvenile red squirrels (*Sciurus vulgaris*) were found with moist, crusty skin lesions. Histology, electron microscopy, and cell culture isolation revealed an orthopoxvirus-like infection. Subsequent PCR and genome analysis identified a new poxvirus (Berlin squirrelpox virus) that could not be assigned to any known poxvirus genera.

The Eurasian red squirrel (*Sciurus vulgaris*) is the only species of tree squirrels endemic throughout most of Europe. Although they are usually abundant, red squirrels are endangered or extinct in some regions in Great Britain and Ireland that are co-inhabited by invasive eastern gray squirrels (*Sciurus carolinensis*), which were introduced from North America in the late 19th century. One major threat is the transmission of squirrelpox virus (SQPV) from the gray squirrel reservoir host to red squirrels, which succumb to lethal infections (*1*). SQPV had been assigned to the parapoxviruses due to morphological similarities (*2*), but the latest viral genome data placed it in a separate clade within the poxvirus family (*3*). Recently, different poxviruses have been associated with similar lesions in American red squirrels (*Tamiasciurus hudsonicus*) from Canada (*4*), but except for a single case report from Spain (*5*), no poxvirus infections in squirrels have been reported in continental Europe.

## The Study

In 2015 and 2016, at least 10 abandoned weak juvenile red squirrels were submitted to a sanctuary near Berlin, Germany. The animals had exudative and erosive-to-ulcerative dermatitis with serocellular crusts at auricles, noses, digits, tails, and genital/perianal regions. Skin specimens from affected animals were investigated by electron microscopy (EM) and PCR. Three animals that died under care were submitted for necropsy. We obtained samples of all organs for histological and PCR examination. We used 1 sample of a skin lesion for virus propagation in cell culture.

**Figure 1:**
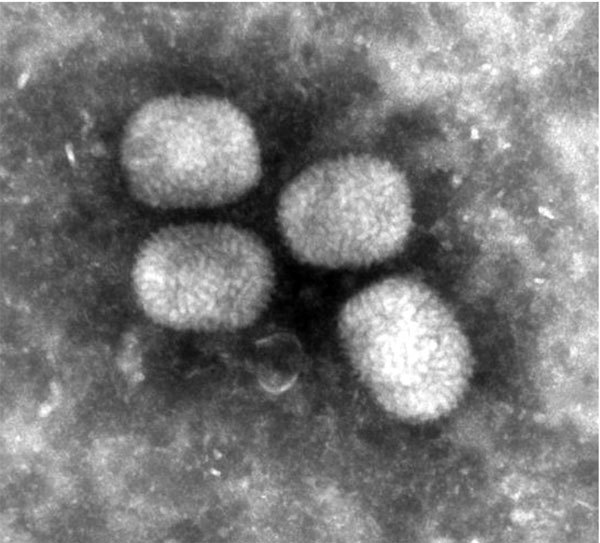
Ultrastructure of Berlin squirrelpox virus particles from skin lesions on a red squirrel in Berlin, Germany, visualized by negative staining (uranyl acetate) (original magnification ×68,000).

EM-negative staining of skin lesions from all animals led to the discovery of brick-shaped poxvirus particles with irregular threadlike surface fibers and an average size of 294 nm × 221 nm (Figure 1). Pathological findings of corresponding skin lesions were consistent with poxvirus infection (ballooning degeneration of epidermal keratinocytes, numerous intracytoplasmic inclusion bodies, epidermal ulceration with suppurative inflammation, and secondary bacterial infection). All inner organs had either no pathological changes or lesions unrelated to poxvirus infection.

To confirm the morphologic diagnosis, we extracted DNA from skin lesions and performed various PCRs. An orthopoxvirus (OPV)–specific PCR showed negative results (*6*); a parapoxvirus (PPV)–specific PCR (*6*), a leporipoxvirus-specific PCR (A. Nitsche and L. Schrick, unpub. data), and a poxvirus-screening PCR (*7*) were positive for some samples. Obtained sequence fragments indicated poxviral relatedness but did not allow for the assignment to a poxvirus genus. Thus, we performed massively parallel sequencing. We directly subjected DNA extracted from a skin lesion on the foot of a dead animal to Nextera XT Library preparation and sequenced it on an Illumina HiSeq 1500 instrument (Illumina, San Diego, CA, USA), yielding 7,242,301 paired-end reads (150 + 150 bases, rapid run mode). Mapping (*8*) the obtained reads to all poxvirus reference sequences available in GenBank in high-sensitivity mode provided no notable results, which pointed to a virus with a highly deviant genome. Therefore, we separated poxviral reads from background data using RAMBO-K version 1.2 (*9*) and assembled the resulting 1,520,811 reads (*10*), yielding 1 single contig of 142,974 bp with ≈460-fold coverage after manual iterative mapping and scaffolding. We confirmed the genomic sequence by resequencing (Illumina MiSeq) of a Vero E6 cell-culture isolate obtained from a different skin specimen of the same animal. We named the new virus Berlin SQPV (BerSQPV), and uploaded the combined sequence information to GenBank (accession no. MF503315). Direct sequencing of DNA from skin samples of three other animals from the same origin yielded sequences with >99.9% identity to BerSQPV.

**Figure 2:**
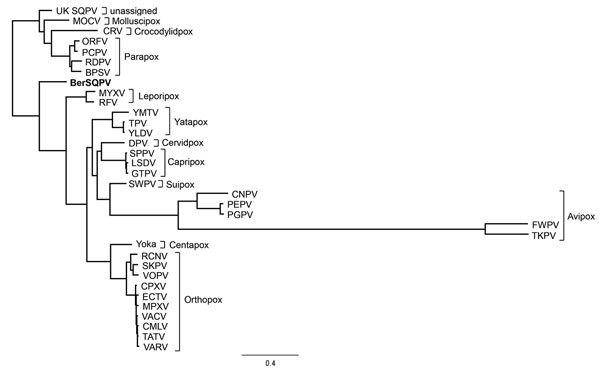
Phylogenetic position of BerSQPV (bold) from a red squirrel in Berlin, Germany, within the *Chordopoxvirinae*. We used MAFFT (*12*) to perform multiple alignments of all complete genome sequences within a species of the *Chordopoxvirinae* subfamily available in GenBank. The minimum pairwise identity found within any of these intraspecies alignments was 79.1%; the maximum pairwise identity of BerSQPV with any chordopoxvirus genome available was 47%. Because of this extreme difference in minimum pairwise identities, we selected individual prototype genomes for each species and the viruses with highest identity to BerSQPV for phylogenetic analysis (as indicated in figure). We performed a multiple alignment of these representative sequences with the BerSQPV genome and removed low-quality regions from the alignment using Gblocks version 0.91(*13*), yielding a stripped alignment of 52,563 gap-free positions. The maximum-likelihood tree was then calculated using PhyML(*14*) (general time reversible plus gamma, 4 substitution rate categories, no invariable sites, BEST topology search, χ^2^-based parametric branch supports). Scale bar indicates nucleotide substitutions per site. BPSV, bovine papular stomatitis virus BV-AR02 (NC_005337); CMLV, camelpox virus CMS (AY009089); CNPV, canarypox virus Wheatley C93 (NC_005309); CPXV, cowpox virus Brighton Red (AF482758); CRV, Nile crocodilepox virus (NC_008030); DPV, deerpox virus W-848–83 (NC_006966); ECTV, ectromelia virus Moscow (AF012825); FWPV, fowlpox virus NVSL (NC_002188); GTPV, goatpox virus Pellor (NC_004003); LSDV, lumpy skin disease virus NI-2490 (NC_003027); MOCV, Molluscum contagiosum virus subtype 1 (NC_001731); MPXV, monkeypox virus Zaire-96-I-16 (AF380138); MYXV, myxoma virus Lausanne (NC_001132); ORFV, Orf virus OV-SA00 (NC_005336); PCPV, pseudocowpox virus VR634 (NC_013804); PEPV, penguinpox virus (KJ859677); PGPV, pigeonpox virus FeP2 (NC_024447); RCNV, raccoonpox virus Herman (NC_027213); RDPV, red deer pox virus (KM502564); RFV, rabbit fibroma virus Kasza (AF170722); SKPV, skunkpox virus (KU749310); SPPV, sheeppox virus 17077–99 (NC_004002); UK SQPV, squirrel poxvirus Red squirrel UK (HE601899); SWPV, swinepox virus 17077–99 (NC_003389); TATV, taterapox virus Dahomey 1968 (NC_008291); TKPV, turkeypox virus HU1124/2011 (KP728110); TPV, tanapox virus (EF420156); FukVACV, vaccinia virus Copenhagen (M35027); VARV, variola major virus Bangladesh-1975 (L22579); VPXV, volepox virus (KU749311); YLDV, Yaba-like disease virus (NC_002642); YMTV, Yaba monkey tumor virus (NC_005179); Yoka, Yokapox virus (NC_015960)].

We compared characteristics of BerSQPV to related viruses and found that the EM structure shows features typical for OPV but the genome size of ≈143 kb is more consistent with PPV or SQPV from the United Kingdom (*11*) than with the large genome of OPV, whereas the guanine-cytosine (GC) content of 38.5% is more consistent with OPV and leporipoxvirus than with PPV and SQPV from the United Kingdom. Therefore, we explored the genomic relationship of BerSQPV to other chordopoxviruses. Pairwise alignments of each of the chordopoxvirus genomes available in GenBank with the BerSQPV genome resulted in a pairwise identity of at most 47% to tanapox virus isolate TPV-Kenya (accession no. EF420156.1). The retrieved phylogenetic tree (Figure 2) demonstrates that BerSQPV cannot be assigned to any of the known poxvirus genera; moreover, it does not cluster with the only other squirrel poxvirus with a published genome sequence (*11*). Further phylogenetic analyses based on conserved single genes frequently used for poxvirus tree calculations (A3L, F10L+F12L, F13L, E13L, E9L [VACV Copenhagen nomenclature]) showed similar results (A56R was not used for tree calculations because this open reading frame is too divergent among the *Chordopoxvirinae*), with BerSQPV forming a unique branch (data not shown). In addition, any partial sequences of SQPV available in GenBank were aligned to BerSQPV, showing a maximal sequence identity of 64.3% to gene E9L (GenBank accession no. AY340976.1), further emphasizing the uniqueness of this newly identified virus.

We designed a BerSQPV-specific quantitative PCR based on the genome sequence as a tool for future investigations (primer BerSQPV_F: ggAAgTTTTCCCATACCAACTgA, primer BerSQPV_R: ATCTCAAACCgCAgACggTA, probe BerSQPV_TM: FAM-ACTgTTATTCTTAgCgTAATT). Sensitivity was <10 genome equivalents per reaction amplifying plasmid dilution rows. We first validated the specificity in silico during the design process, revealing the highest identity of 88% to cowpox virus Kostroma (GenBank accession no. KY369926.1), with mismatches in crucial positions in the primer and probe binding sites. Squirrel poxvirus strain Red squirrel UK (GenBank accession no. NC_022563.1) showed only 84% identity, with additional mismatches in amplification-relevant positions. Practical PCR testing using DNA from cowpox, monkeypox, ectromelia, parapox-ORF, myxoma, avipox, and molluscipox viruses showed no cross-reactivity. 

The new specific quantitative PCR was subsequently applied to DNA from skin lesions archived from 1 squirrel found dead in 2014 in the Berlin area, 2 live squirrels from 2015, and 5 live squirrels from 2016, as well as various organs from 3 affected squirrels necropsied in 2015 (Table). Organ tissues yielded high BerSQPV DNA loads in the affected skin but low viral DNA loads for inner organs, findings in concordance with pathological findings, indicating the detection of viral DNA in the blood homogenously distributed throughout the organs with specific tropism for the skin. Low virus loads in inner organs are usually observed in poxvirus infections that do not generalize. PCR results indicate that this virus has been circulating in the Berlin area over the past 10 years.

**Table:** Results of PCRs of different tissues from 7 live and 4 deceased squirrels showing poxvirus lesions, Berlin, Germany*

Year of sampling	Live/dead	Tissue	Cq BerSQPV	Cq c-myc	ΔCq (BerSQPV − c-myc)
2014	Dead	Archived skin (paraffin)	23.7	34.8	−11.1
2015	Live	Crust‡	12.5	17.7	−5.2
2015	Live	Crust‡	14.8	18.3	−3.5
2015	Dead	Skin (foot)‡	11.1	18.8	−7.7
		Skin (tail)	9.7	17.9	−8.2
		Skin (toe)†‡	10.1	18.6	−8.5
		Lung	33.2	27.0	6.2
		Liver	34.7	23.1	11.6
		Spleen	34.9	23.9	11.0
		Brain	33.9	24.5	9.4
2015	Dead	Skin (forefoot)‡	10.9	18.2	−7.3
		Skin	26.3	28.0	−1.7
		Lung	33.6	23.1	10.5
		Liver	Negative	22.1	NA
		Spleen	38.3	23.9	14.4
		Kidney	Negative	24.1	NA
		Small intestine	Negative	21.8	NA
		Large intestine	Negative	24.4	NA
		Brain	Negative	25.3	NA
2015	Dead	Crust	19.0	23.2	−4.2
		Lung	35.2	25.4	9.8
		Liver	Negative	20.8	NA
		Spleen	34.0	25.1	8.9
		Kidney	Negative	25.9	NA
		Small intestine	36.4	21.6	14.8
		Large intestine	35.0	23.5	11.5
		Brain	Negative	24.6	NA
2016	Live	Crust	15.0	22.0	−7.0
2016	Live	Crust	12.1	18.6	−6.5
2016	Live	Crust	14.1	20.8	−6.7
2016	Live	Crust	13.2	17.7	−4.5
2016	Live	Crust	12.9	18.3	−5.4

*BerSQPV DNA was quantified in relation to cellular c-myc DNA; lower values for ΔCq indicate higher virus loads in a respective tissue. BerSQPV, Berlin squirrelpox virus; Cq, quantification cycle; NA, not applicable. †Specimen applied to next-generation sequencing ‡Specimen used to obtain the cell culture isolate

## Conclusions

We describe a new poxvirus, BerSQPV, isolated from red squirrels in Berlin, Germany, that causes pathological changes consistent with other epidermal poxvirus infections. Genome analysis revealed a unique sequence within the poxvirus family, as BerSQPV is not clustering to other poxvirus genera, including UK SQPV from red squirrels from Great Britain. In contrast to UK SQPV, which resembles PPV ultrastructurally (*2*), the ultrastructure of BerSQPV is comparable to that of OPV. Two other poxviruses from tree squirrels with ultrastructural appearance similar to BerSQPV have been reported: a Eurasian red squirrel from Spain with epidermal poxvirus lesions (*5*) and American red squirrels from Canada (*15*). Although no sequence information is available for the SQPV from Spain, the partial sequence analysis of SQPV from Canada showed the virus to also be distinct from all known mammalian poxviruses but most closely related to PPV, followed by UK SQPV (*15*). 

BerSQPV is suspected to have been circulating for several years among Eurasian red squirrels in the greater Berlin area. Although diseased animals in care were handled in close contact, caretakers have remained asymptomatic, suggesting a negligible risk for human infection. Further detailed characterization of the isolated virus is ongoing.
